# Effect of Obesity in Persistent or Remission in Postmenopausal Women with Atypical Cervical Cytology 

**DOI:** 10.31557/APJCP.2019.20.12.3783

**Published:** 2019

**Authors:** Paweena Phaliwong, Wiyada Luangdansakul, Kornkarn Bhamarapravatana, Komsun Suwannarurk

**Affiliations:** 1 *Reproductive Medicine Unit, Department of Obstetrics and Gynecology, Bhumibol Adulyadej Hospital, *; 2 *Department of Preclinical Science, *; 3 *Gynecologic Oncology Unit, Department of Obstetrics and Gynecology, Faculty of Medicine, Thammasat University Hospital, Bangkok, Thailand. *

**Keywords:** ASC-US, CIN, menopause, obesity

## Abstract

**Objectives:**

To determine the effects of obesity in pre and postmenopausal women diagnosed with atypical squamous cells of undetermined significance (ASC-US) (in cases of remission, persistence, and progression of disease) after initial management and follow-up within 2 years to inform proper management for postmenopausal Thai women.

**Methods:**

This retrospective study was conducted at Bhumibol Adulyadej Hospital, Thailand between January 2013 and October 2018. Medical records of 506 patients whose cervical cytology reported of ASC-US were reviewed. Prevalence of silent high grade cervical intraepithelial neoplasia (CIN2/3) was evaluated. Cervical cytology after completed follow-up within 2 years were determined.

**Results:**

During the study period, there were 506 cases of ASC-US cytology reported. One quarter of cases were of post-menopausal status. Prevalence of CIN 2/3 in ASC-US cytology in pre and postmenopausal women were 9.9 and 7.2%, respectively. At 2-year follow-up appointments, around 90% of patients who completed follow-up remained in remission of the disease in both age groups. Menopausal status, body mass index (BMI), sexual activity, number of sexual partners, parity, smoking and hormone replacement therapy were not correlating factors to remission. Obese postmenopausal women had a lower remission rate of CIN, but this finding was not statistically significant.

**Conclusion:**

Obesity was not found to correlate strongly with the progression or remission of CIN. Obese postmenopausal women may still be affected by a persistence of the disease. Continuing cervical cytology monitoring should be recommended for such patients. Silent high grade cervical intraepithelial neoplasia (CIN2/3) in ASC-US cytology in this study were high. Colposcopy should be recommended for diagnosis and follow-up in this setting.

## Introduction

Menopause is the permanent cessation of menses, resulting from the reduced secretion of ovarian hormones namely estrogen and progesterone. This takes place as the finite store of ovarian follicles are depleted (Al-Safi et al., 2015). Thai women typically begin menopause at an age range of 48-52 years, with a median age of 51 years (Ongsupharn et al., 2018).

Cervical cancer screening is a simple tool that could be used to detect precancerous and cancerous cervical lesions. In many studies, it has been found that the most common cervical cytology is the atypical squamous cells of undetermined significance (ASC-US). This was shown in many studies in Thailand and other Southeast Asian countries (Laiwejpithaya et al., 2008; Tanabodee et al., 2015; Kingnate et al., 2016; Mu-Mu-Shwe et al., 2014; Hav et al., 2016). It is known that the cervical cytology of the ASC-US type is an atypical report that indicated either a low-risk or high grade cervical cytology abnormality. Therefore, treatment was still not a definite conclusion (Massad et al., 2013).

In 2012, the American Society for Colposcopy and Cervical Pathology (ASCCP) had revised the consensus guidelines for the management of abnormal cervical cancer screening tests and cancer precursors. According to ASCCP 2012, the acceptable management in women with ASC-US on cytology was either a repeat cytology at one year or an immediate human papillomavirus (HPV) testing (reflex test). Colposcopy was recommended in ASC-US cases with positive result of high risk HPV test (Massad et al., 2013).

Literatures showed that the prevalence of ASC-US cytology in menopausal women was 1.8 percent (Keating et al., 2001; Cakmak et al., 2014). Incidence of silent cervical intraepithelial neoplasia (CIN) 2/3 in menopausal women ranged from 1.8 to 6.1 percent (Tokmak et al., 2014; Goksedef et al., 2011).

The objective of this study was to evaluate the effect of obesity and BMI on remission, persistence and progression of the disease in pre and postmenopausal women with a cytology of ASC-US after initial management and a 2 year follow up. The prevalence of silent high grade cervical intraepithelial neoplasia (CIN 2/3 and cancer) in ASC-US cytology results in both groups of patients were studied.

## Materials and Methods

This was a retrospective study, approved by the institutional review board, Bhumibol Adulyadej Hospital (BAH), Bangkok, Thailand (IRB: 18/62). Medical records stored in computerized systems were reviewed. Data from all patients with cervical cancer screening data in a 6 year period between January 2013 and October 2016 were enrolled. Inclusion criteria were women who had a cervical cytology result with ASC-US cytology. Exclusion criteria included pregnancy, hysterectomy and known histories of pre-invasive or invasive cervical lesions or other gynecologic cancers. Papanicolaou smears were performed using conventional (CPP) or liquid based cervical cytology (LBP) and evaluated according to the 2001 Bethesda. Subjects were then divided into pre and postmenopausal women groups. Demographic characteristics consisted of age, menstrual status, parity, screening proposal, history of sexually transmitted diseases (STD) including human immunodeficiency virus (HIV), number of sexual partners, smoking, alcohol consumption and education. Management of ASC-US cytology was followed by the previous ASCCP guideline by either a repeated cervical cytology testing in 6 months or a colposcopic directed biopsy (immediate colposcopy or positive high risk HPV test). Outcomes were classified as remission, persistent disease, completed and loss to follow-up after the initial of ASC-US management at a two year follow up appointment. Remission was defined as normal cervical cytology results at follow-up within two years. Persistent disease was defined as abnormal cervical cytology and colposcopic directed biopsy results at follow-up within two years. Completed and loss to follow-up were defined as a completion of cervical cytology or an absence of follow-up within two years. 

The data was analyzed by using the SPSS statistical software version 18 (IBM, Armonk, NY, USA) for analysis. Descriptive statistics were used to analyze patient demographic data. Continuous variables were presented as mean and standard deviation (SD). Categorized data were expressed as number and percentage. Pearson Chi-square and Fisher’s exact test were used in data analysis when appropriate. The p-value of less than or equal to 0.05 was considered to be statistical significance. Multivariable analysis of patient risk factors was calculated using binary logistic regression analysis and described as an odds ratio.

## Results

This study was conducted in the main hospital of the Royal Thai Air Force. In this study, around ninety percent of the premenopausal women were air force officers and employees while fifty percent of postmenopausal women were housewives. Participants were people who worked at and lived in the surrounding area of the hospital. 

**Figure 1:**
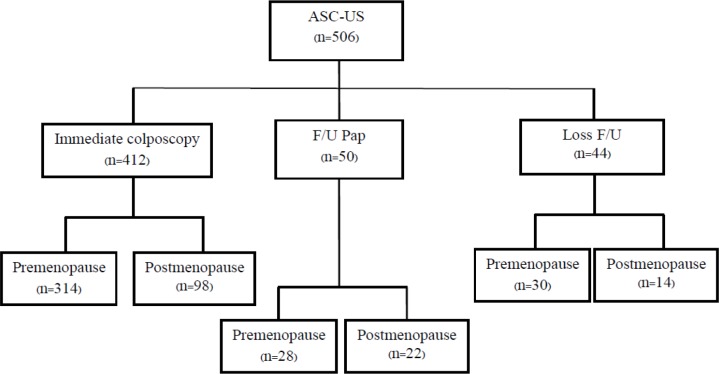
Medical Record Reviewed in This Study. ASC-US, atypical squamous cells of undetermined significance; F/U, follow up

A total of 506 patients with ASC-US cytology were enrolled. Subjects underwent either immediate colposcopy or follow up cervical cytology after thorough counseling, at an incidence of 412 and 50 cases, respectively (Figure 1).

The mean age of women in premenopausal versus postmenopausal women groups were 37.4 and 61.1 years, respectively. The participants in postmenopausal women had higher mean age of coitarche than premenopausal women with statistically significance (19.8 vs 17.9, p< 0.001). Ninety-seven percent of cases in the postmenopausal women group were still sexually active.

Demographic data of subjects with ASC-US reports are presented in Table 1. There were 372 and 134 cases in pre and postmenopausal women group, respectively.

Half of the postmenopausal group was classified as unsatisfactory colposcopy (Type 3). It was more than those of the premenopausal women group with statistical significance as shown in Table 2. The histopathological results showed the prevalence of less than or equal to CIN 1 and CIN 2/3 in ASC-US cytology at 90.1/92.8 and 9.9/7.2 percent in pre and postmenopausal women groups, respectively. Women with conclusive results for CIN 2/3 underwent Cone biopsy either by either Loop Electrosurgical Excision Procedure (LEEP) or Cold Knife Conization (CKC) performed in those who had cervical biopsies of CIN 2/3. Neither micro invasive or invasive cervical cancer was found in both groups.

At two year follow-up periods, in immediate colposcopy arm, 233 patients (56.6%) had completed follow-up. Among non-obese cases, women in postmenopausal group had remission rate lower than premenopausal group with statically significant (91 vs 99 percent). Among obese women, remission rate of pre and postmenopausal women were equal. Among patients who chose follow up cervical cytology, remission rate of pre and postmenopausal women of the obese and non-obese categories were comparable. No progressive disease was shown in both groups including both management groups as presented in Table 3.

The multivariate analysis of correlation of the remission disease and clinical factors (including menopausal status, body mass index (BMI), sexual activity, parity, smoking and hormone replacement therapy) was performed as shown in Table 4. There was no significant correlation between clinical factors and the progression or regression of disease.

**Table 1:** Baseline Characteristics of Women Participating in This Study

Characteristics	Pre	Post	*P-value*
	(n=372)	(n=134)	
Age (years) *	37.4 + 9.4	61.1 + 7.5	< 0.001***
BMI (kg/m^2^)*	22.7 + 4.1	25.5 + 4.6	< 0.001***
Coitarche (years) *	17.9 + 7.7	19.8 + 4.6	0.001***
Sexual intercourse**	322 (86.6)	130 (97.0)	<0.001***
Monogamy**	340 (91.4)	119 (88.8)	0.375
Parity**			<0.001***
0-1	258 (69.4)	52 (38.8)	
≥ 2	114 (30.6)	82 (61.2)	
Screening proposal**			0.027
Check up	306 (82.3)	121 (90.3)	
Others	66(17.7)	13 (9.7)	
Non smoking**	368 (98.9)	132 (98.5)	0.658
Alcohol consumption**	6 (1.6)	2 (1.5)	1
HIV positive**	4 (1.1)	0 (0)	0.577
Education**			0.001***
Below bachelor	108 (29.0)	60 (44.8)	
Bachelor or higher	264 (71.0)	74 (55.2)	
LBP type**	104 (28.0)	24 (17.9)	0.022***
Specimen adequacy**	190 (51.1)	56 (41.8)	0.065
BMI level			< 0.001***
Normal	222 (59.7)	46 (34.3)	
Overweight	61 (16.4)	18 (13.4)	
Obese	89 (23.9)	70 (52.3)	
Medical disease			< 0.001***
No	352 (94.6)	81 (60.4)	
Yes	20 (5.4)	53 (39.6)	

*: mean + SD (standard deviation), **: n (%), ***: The result is significant at p <0.05, Pre: premenopausal women, Post: postmenopausal women, HIV: human immunodeficiency virus, LBP: liquid based cervical cytology, BMI: body mass index, Normal:

**Table 2:** Results of Pre- and Postmenopausal Women who had Cytology of ASC-US

Results	Premenopause (n=314)	Postmenopause (n=98)	*p-value*
Colposcopic findings**			< 0.001***
Type 1/2	232 (73.9)	46 (46.9)	
Type 3	82 (26.1)	52 (53.1)	
Histopathologic results**			0.4
≤ CIN 1	283 (90.1)	91 (92.8)	
CIN 2/3	31 (9.9)	7 (7.2)	

**: n (%), ***: The result is significant at p<0.05, ASC-US: atypical squamous cells of undetermined significance, ≤ CIN 1: cervical intraepithelial neoplasia grade 1 or less, CIN 2/3: cervical intraepithelial neoplasia grade 2 and 3.

**Table 3:** Results of Pre- and Postmenopausal Women who had Completed a 2-Year F/U

Characteristics	Non-obese		*p-value*	Obese		*p-value*
	Pre	Post		Pre	Post	
IC Arm			0.010***			0.369
Remission	115 (99.1)	32 (91.4)		47 (95.9)	33 (100)	
Persisted	1 (0.9)	3 (8.6)		2 (4.1)	0 (0)	
F/U Arm			0.505			0.472
Remission	15 (100)	11 (100)		11 (100)	9 (90)	
Persisted	0 (0)	0 (0)		0 (0)	1 (10)	

Pre: premenopausal women, Post: postmenopausal women, IC: immediate colposcopy, F/U: follow up, Remission: remission of disease, Persisted: persistent of disease, Non-obese: BMI 18.5-29.9 kg/m^2^, Obese, BMI > 30 kg/m^2^; ***: The result is significant at p<.05

**Table 4:** Correlation of the Remission of Disease and Patient Factors by Multivariate Analysis

Factors	Remission	Persisted	OR (95% CI)	*P-value*
Menopausal status				
Premenopause	188	3	3.12 (0.57-17.09)	0.189
Postmenopause	75	3	0.31(0.05-1.64)	0.169
BMI				
Normal	128	3	1.01 (0.18-5.61)	0.983
Overweight	40	0	3.03	0.998
Obese	95	3	0.92 (0.17-4.92)	0.922
Sexual intercourse				
No	31	0	2.24	0.998
Yes	232	6	0	0.998
Number of sexual partners				
0-1	237	6	0	0.998
≥ 2	26	0	5.36	0.998
Parity				
0-1	171	3	1.97 (0.34-11.26)	0.445
≥ 2	92	3	0.51 (0.08-2.89)	0.445
Smoking				
No	260	6	2.78	1
Yes	3	0	0.23	1
HRT used				
No	259	6	0	0.999
Yes	4	0	1.16	0.999

BMI, body mass index; Normal, BMI 18.5-22.9 kg/m^2^; Overweight, BMI 23-29.9 kg/m^2^; Obese, BMI > 30 kg/m^2^; HRT, hormone replacement therapy; Remission, remission of disease; Persisted, persistent of disease; OR, odds ratio; CI, confidence interval; **, The result is significant at p < 0.05

## Discussion

ASC-US is the most common cytological abnormality in cervical cytology. It carried the lowest risk of CIN 3 or cancer because one third to two third was not HPV associated (Katki et al., 2013).

Age of coitarche in the premenopausal group was lower than that of the postmenopausal group (17 and 19 years, respectively). These findings might be the result of different occupations and life styles among both groups in this study. Half of the postmenopausal group were housewives while eighty percent of premenopausal group were employees in either government or the private sector.

Most of both groups were monogamous, non-smokers and non-consumers of alcohol (in line with demographic trends of Thai women). However, the effect of the second hand cigarette smoke could not be evaluated.

In the current study, mean BMI of the postmenopausal women was higher than that of the premenopausal women (25.5 vs 22.7 kg/m^2^). Abnormal BMI level indicated overweight and obese status. Two third of postmenopausal women in the present study had abnormal BMI. Fifty percent of this group had BMI in obese ranges. The findings were comparable to the results from Chen KL et al that reported increasing prevalence of overweight and obesity in 70 percent of postmenopausal women (Chen et al., 2018). 

From the present study, postmenopausal women were more likely to be obese and have underlying diseases including diabetes mellitus, hypertension and cardiovascular disease, similar to the findings reported in previous literature (Krychman et al., 2015). According to 2003-2012 National Health and Nutrition Examination Survey (NHANES) data, metabolic syndrome presented in about 34 percent of the population and the rates of metabolic syndrome were increasing among postmenopausal women (Chen et al., 2018). Among postmenopausal women in this study, there was a forty percent incidence of metabolic disease. The rate of findings was comparable to the previous literature.

The rate of inadequate colposcopy in postmenopausal women in this investigation was significantly higher than that of the premenopausal women (53.1 and 26.1 %, respectively). This finding was a consequence of physiologic migration of the squamocolumnar junction (SCJ) to the cervical opening. Local estrogen therapy to the cervicovaginal region improved the adequacy of colposcopic examination (Goksedef et al., 2011; Richards et al., 2015).

In the current study, the prevalence of silent CIN2/3 in ASC-US cytology were 9.9 and 7.2 percent in pre and postmenopausal groups, respectively. Both groups had high prevalence of high grade CIN. Thus we recommended that all women with ASC-US cytology results should undergo colposcopic directed biopsy. Our recommendation differed from the American Society for Colposcopy and Cervical Pathology (ASCCP) guidelines that recommended colposcopy in persistent ASC-US cytology or concomitant with positive high risk HPV (Massad et al., 2013). 

Among non-obese cases in both pre and postmenopausal groups, persistence rates of abnormal cervical cytology of the postmenopausal group was higher than that of the premenopausal group (3/35 and 1/116, respectively). Using binary logistic regression analysis, no statistically significant links were found between remission rates of abnormal cervical cytology and patient factors (including menopausal status, BMI, sexual activity, parity, smoking and HRT). Among postmenopausal women with obese BMIs, there was a slightly lower rate of disease regression, but this was found to not be statistically significant. This was explained by the alteration of host immunity (Liu et al., 2013). Age was an important risk factor for a persistent HPV infection due to decreasing immune function in the elderly. HPV was hard to be clear after its acquisition (Sui et al., 2018). 

Obesity was a result of disruption of energy balance that led to weight gain and metabolic disturbances that causes tissue stress and dysfunction. Metabolic disturbances lead to immune activation in tissue such as adipose tissue, liver, pancreas and vasculature. These effects may increase the risk for other infectious and chronic diseases (Andersen et al., 2016). However menopause was often accompanied by redistribution of adipose tissue from the periphery to the abdomen, hip and thigh. Central obesity has been associated with a pro-inflammatory state in postmenopausal women. Adiposity had been linked to increased susceptibility to viral pathogens, such as herpes simplex virus-1 and -2 and adenovirus-36 (Liu et al., 2013). In particular, Baker et al (Baker et al., 2011) showed increased levels of adipokines in older women with persistent HPV infection. 

The limitations of this study were its nature as a retrospective study. Demographic data and further follow-ups of cervical cytology were not completed. 

There was no data on blood hormonal, adipokine and immunological factor levels in our retrospective data. It would cause of remission or persistence of disease in women with BMI status in obese level between pre and postmenopausal women with no difference. Therefore, this topic should be of concern and merits further study. 

In conclusion, in postmenopausal women, obesity might have a link on the remission rate. Cervical cytology follow-up should be prescribed in this population. However, obesity was not correlated with progression or regression of CIN, and further studies should be done to clarify the relationship. The prevalence of silent high grade CIN in ASC-US cases in pre and postmenopausal women were higher than the data of ASCCP guideline. Based on our patient population, we recommended immediate colposcopy in all women who had cervical cytology report of ASC-US without HPV testing result. The suggestion might be applied to the referral centers with high incidence of silent high grade CIN. 

## References

[B1] Al-Safi ZA, Polotsky AJ (2015). Obesity and menopause. Best Pract Res Clin Obste Gynaecol.

[B2] Andersen CJ, Murphy KE, Fernandez ML (2016). Impact of obesity and metabolic syndrome on immunity. Adv Nutr.

[B3] Baker R, Dauner JG, Rodriguez AC (2011). Increased plasma levels of adipokines and inflammatory markers in older women with persistent HPV infection. Cytokine.

[B4] Cakmak B, Köseoğlu DR (2014). Comparison of cervical cytological screening results between postmenopausal and elderly women. Turk Patoloji Derg.

[B5] Chen KL, Madak-Erdogan Z (2018). Estrogens and female liver health. Steroids.

[B6] Goksedef BP, Akbayir O, Baran SY (2011). Atypical squamous cells of undetermined significance in postmenopausal women: a comparative retrospective analysis. Eur J Obstet Gynecol Reprod Biol.

[B7] Hav M, Eav S, Heang N (2016). Prevalence of abnormal cervical cytology in HIV negative women participating in a cervical cancer screening program in Calmette Hospital, Cambodia. Asian Pac J Cancer Prev.

[B8] Katki HA, Schiffman M, Castle PE (2013). Five-year risks of CIN 3+ and cervical cancer among women with HPV testing of ASC-US Pap results. J Low Genit Tract Dis.

[B9] Keating JT, Wang HH (2001). Significance of a diagnosis of atypical squamous cells of undetermined significance for Papanicolaou smears in perimenopausal and postmenopausal women. Cancer.

[B10] Kingnate C, Tangjitgamol S, Khunnarong J (2016). Abnormal uterine cervical cytology in a large tertiary hospital in Bangkok metropolis: Prevalence, management and outcomes. Indian J Cancer.

[B11] Krychman ML (2015). Obesity and sexual function. Menopause.

[B12] Laiwejpithaya S, Rattanachaiyamont M, Benjapibal M (2008). Comparison between Siriraj liquid-based and conventional cytology for detection of abnormal cervicovaginal smears: a split-sample study. Asian Pac J Cancer Prev.

[B13] Liu SH, Rositch AF, Viscidi RP (2013). Obesity and human papillomavirus infection in perimenopausal women. J Infect Dis.

[B14] Massad LS, Einstein MH, Huh WK (2013). 2012 updated consensus guidelinesfor themanagement of abnormal cervical cancer screening tests and cancer precursors. J Low Genit Tract Dis.

[B15] Mu-Mu-Shwe, Harano T, Okada S (2014). Prevalence of high-risk human papillomavirus (HR-HPV) infection among women with normal and abnormal cervical cytology in Myanmar. Acta Med Okayama.

[B16] Ongsupharn S, Pantasri T, Lattiwongsakorn W (2018). The Association between oligomenorrhea, onset of menopause and metabolic syndrome in thai postmenopausal women. J Menopausal Med.

[B17] Richards A, Dalrymple C (2015). Abnormal cervicovaginal cytology, unsatisfactory colposcopy and the use of vaginal estrogen cream: an observational study of clinical outcomes for women in low estrogen states. J Obstet Gynaecol Res.

[B18] Sui S, Zhu M, Jiao Z (2018). Prognosis and related factors of HPV infections in postmenopausal Uyghur women. J Obstet Gynaecol.

[B19] Tanabodee J, Thepsuwan K, Karalak A (2015). Comparison of efficacy in abnormal cervical cell detection between liquid-based cytology and conventional cytology. Asian Pac J Cancer Prev.

[B20] Tokmak A, Guzel AI, Ozgu E (2014). Clinical significance of atypical squamous cells of undetermined significance in detecting pre invasive cervical lesions in post- menopausal Turkish women. Asian Pac J Cancer Prev.

